# Supplementary material for: Massively parallel reporter perturbation assays uncover temporal regulatory architecture during neural differentiation
Source: Nat Commun. 2022 Mar 21;13:1504. doi: 10.1038/s41467-022-28659-0 (PMC8938438; doi:10.1038/s41467-022-28659-0)
Supplement: Supplementary file 1 — Supplementary Information [file 41467_2022_28659_MOESM1_ESM.pdf]

Supplementary Information

Kreimer, Inoue, Ashuach *et al.*

Massively parallel reporter perturbation assays uncover temporal regulatory architecture during neural differentiation

Supplementary Notes

Supplementary Note 1.

Supplementary Figures

Supplementary Figs. 1-13.

Supplementary Tables

Supplementary Tables. 1-5.

Supplementary References

Supplementary Notes

Supplementary Note 1: Determinants of time-point specific regulatory activity.

One of the open questions in Biology is to understand which regulatory elements play a role under different cellular conditions and what determines this cell-type specificity. The answers for these questions can guide designs of synthetic enhancers for the purpose of cell therapy and can help in refining drug design. In our model system, we examined if we can gain a better understanding on the determinants of time-point specific regulatory activity. Cooperative binding of pluripotent factors and neural factors (POU, SOX, NANOG) are known to play important roles. Especially SOX2 and ClassV POU (POU5F1) function as a pioneer factor in ESCs, while other members of SOX and POU (SOX1/2 and POU3F1 etc.) play important roles in NPCs. However, their function depends on genomic context and molecular function of other factors (e.g. OTX2) that play roles in neural induction and are largely unknown.

When we perturb an essential motif, by definition, the enhancer is no longer functional in any of the time points, suggesting that these motifs are required for transcription, but not necessarily for determining a specific temporal pattern of the transcription. We were intrigued to see if we could find such condition specific binding motifs in our data. To that end, we looked for FRSs with motifs in (i) late response WT regions (WT alpha - cluster 3; **Supplementary Fig. 12a**) that exhibit their highest perturbation effect in the later time points (Log2FC cluster 3; **Supplementary Fig. 12a**) and specifically, show significant perturbation effects in 72hr NPC state but not in 0hr embryonic stem cell (ESC) state ; (ii) early response WT regions (WT alpha - cluster 1; **Supplementary Fig. 12a**) that exhibit the highest perturbation effect in the early time points (Log2FC cluster 1; **Supplementary Fig. 12a**) and specifically, show significant perturbation effects in 0hr but not in 72hr. We find 37 sequences that are candidates for driving NPC state (i) and 7 sequences that are candidates for driving ESC state (**Supplementary Dataset 4**). Our premise was that if such motifs are condition (time point) specific they should determine the regulatory activity of a genomic sequence to be in ESC or NPC state. We thus look for enrichment (hypergeometric test FDR<0.05) of such motifs in all 591 WT regions, either in ESCs WTs (cluster 1) or NPC WTs (cluster 3), bearing in my that this is an underpowered test, we only find the following motifs enriched in NPC WT regions: RELA_M4497_1.02 - GGGGATTTCCA, RELB_M6448_1.02 - GGGGGATTTCCA, SP8_1 - GCCACGGCCACT and no motifs were found to be enriched in ESC WT.

Moreover, we find instances where the same motif sequence: e.g., GGGGATTTCCA motif for RELA_M4497_1.02 in region chr1:33808004-33808175 shows functional activity in both NPC (72hr) and ESC (0hr) states. These results suggest that the motif sequence alone is less likely to determine temporality without the context of the surrounding region and other bound factors which have an effect on that.

Interestingly, we observe instances where a similar motif appears more than one time in the same region and has different condition specific effects: CTTTGGATGACAAAGG motif does not show NPC specific effect whereas TTTGGATGACAAAGG and TTGGATGACAAAGG motifs do (SOX1 example; **Supplementary Fig. 12b**), this suggests that there might be specific cases where within the same region we can dissect specific bases that affect temporality.

Supplementary Figures

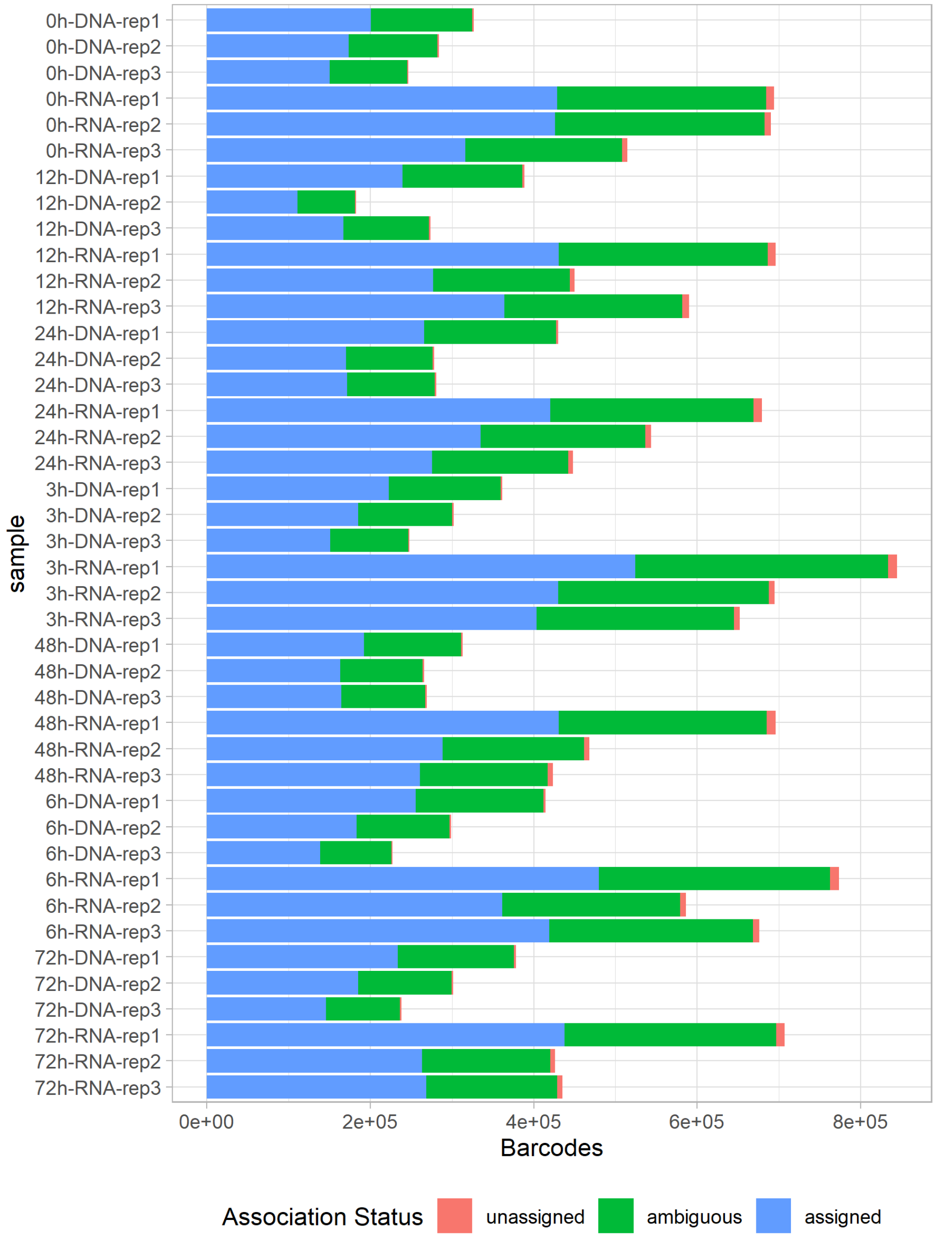

Supplementary Fig. 1: Barcode association status per sample. Source data are provided as a Source Data file.

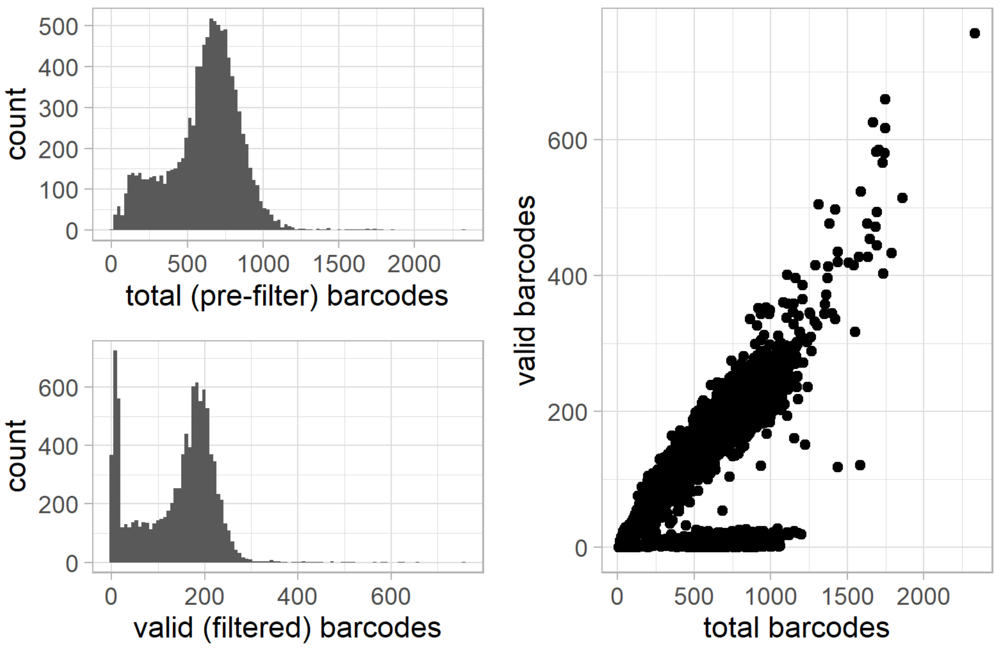

Supplementary Fig. 2: Barcodes per sequence per replicate – pre- and post-filtering. Source data are provided as a Source Data file.

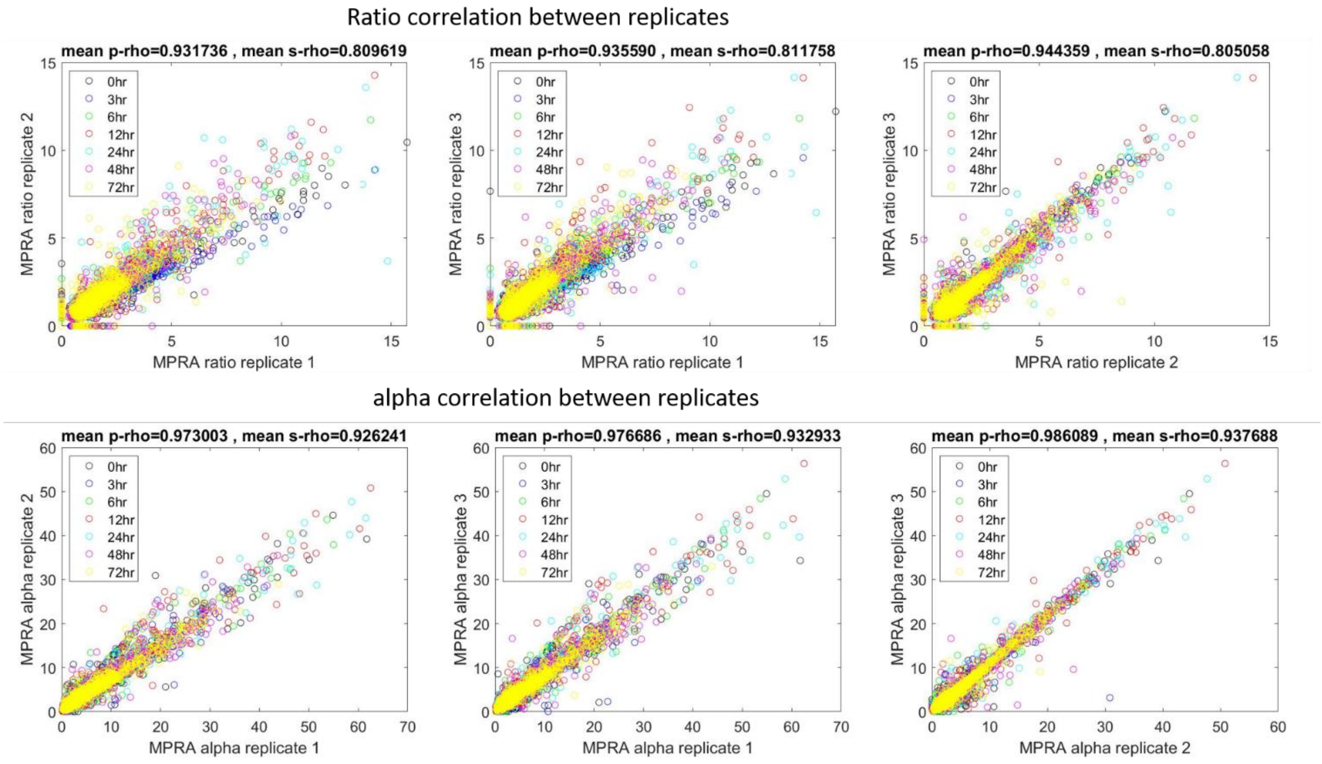

Supplementary Fig. 3: Correlation between replicates signal - ratio (upper panel) and alpha (bottom panel). Source data are provided as a Source Data file.

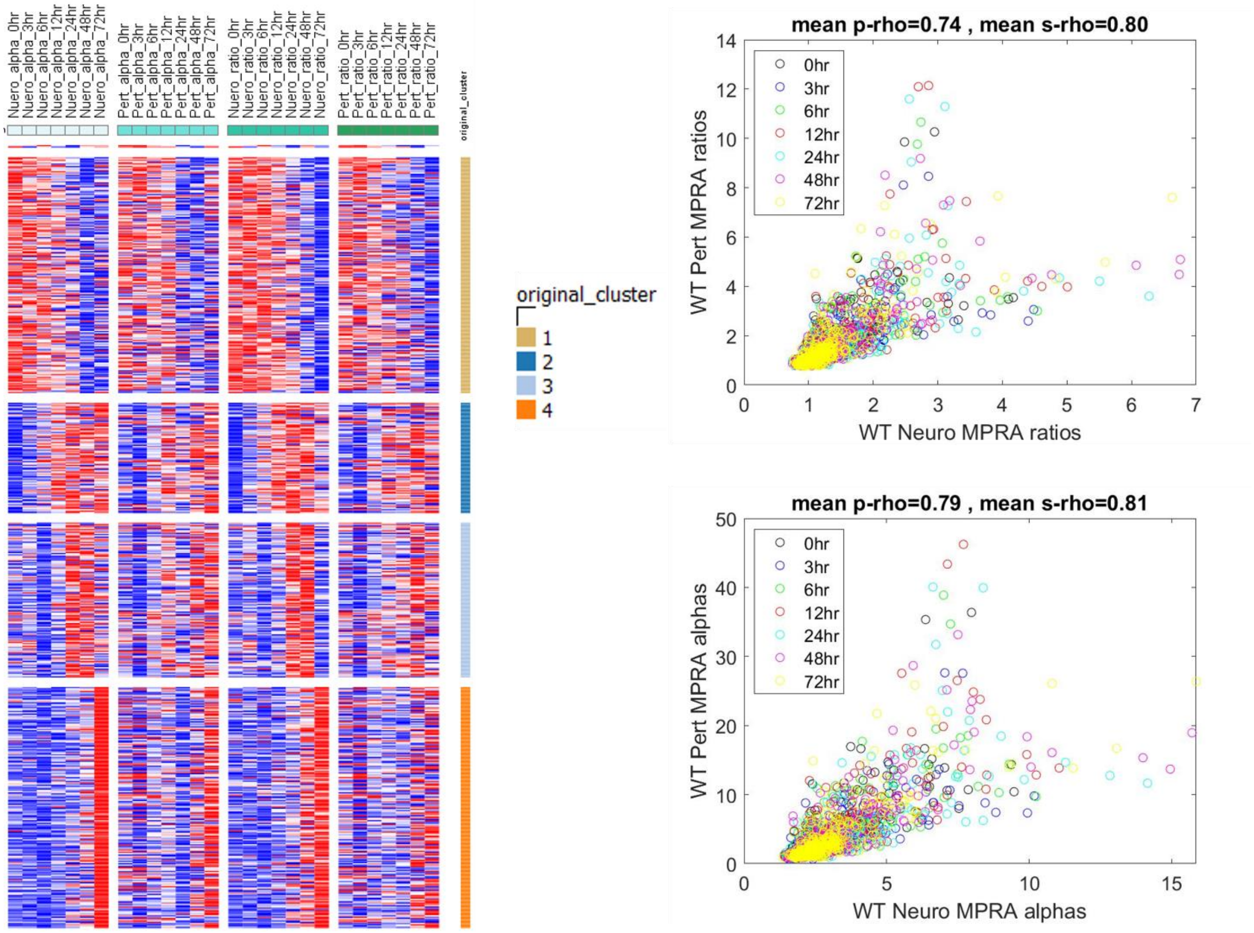

Supplementary Fig. 4: Reproducibility with Inoue *et al.* 2019 [1]. The left panel shows alpha (from MPRAnalyze) and ratio (RNA/DNA) for WT regions – comparing the original data in Inoue *et al.* 2019 (labeled 'Neuro') vs. the current paper (labeled 'Pert'). Each row shows normalized values – ranged from the lowest (blue) to the highest (red). The data is clustered based on the original MPRA clusters from Inoue *et al.* 2019. The right panel shows Pearson and Spearman correlations in each of the seven time points for both ratio (upper) and alpha (bottom) for WT regions between the two experiments. Source data are provided as a Source Data file.

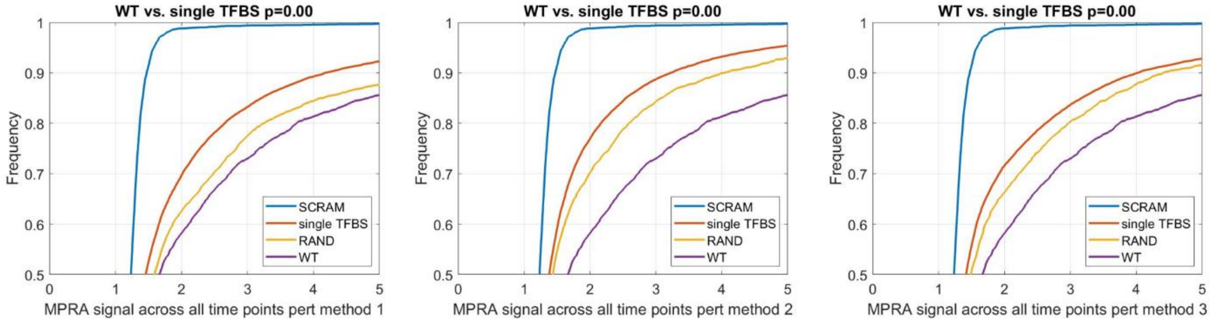

Supplementary Fig. 5: Cumulative distribution of the MPRA signal (alpha) across different categories for the three perturbation methods. Source data are provided as a Source Data file.

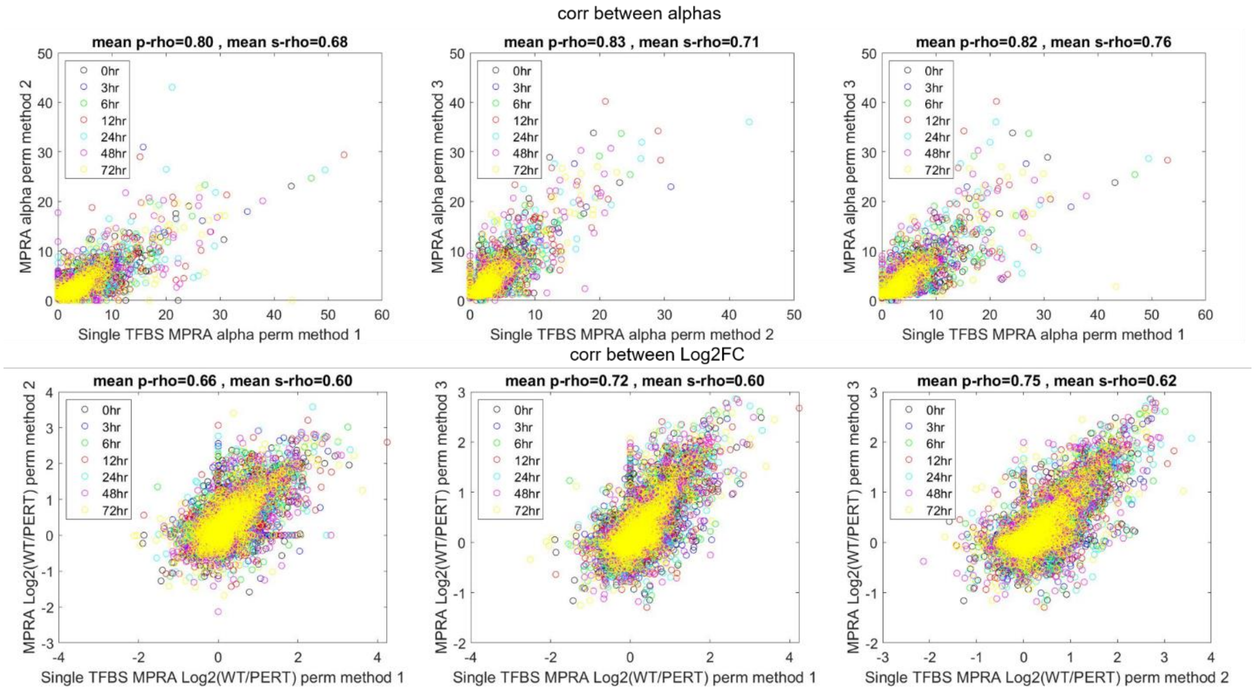

Supplementary Fig. 6: Correlation of alpha (upper panel) and Log2FC (bottom panel) per time point, between the different perturbation methods. Source data are provided as a Source Data file.

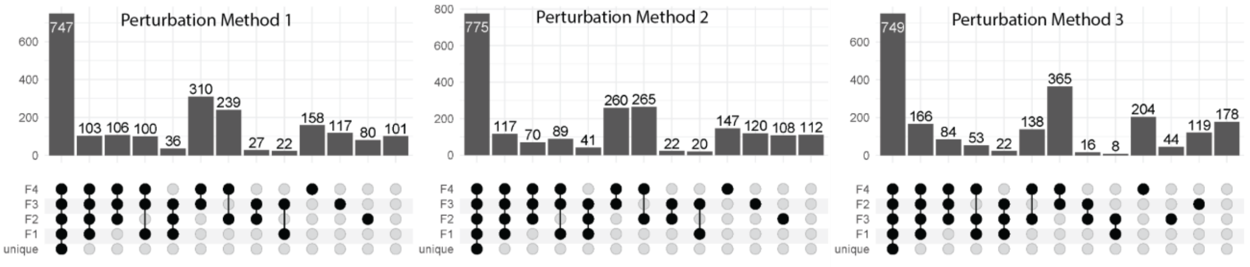

Supplementary Fig. 7: Plots showing the number of tested sequences that pass each filter, with each perturbation method. Histograms show number of sequences counted for each combination of filters, bottom panels describe each combination (filled dot indicates passing the filter, empty otherwise). Source data are provided as a Source Data file.

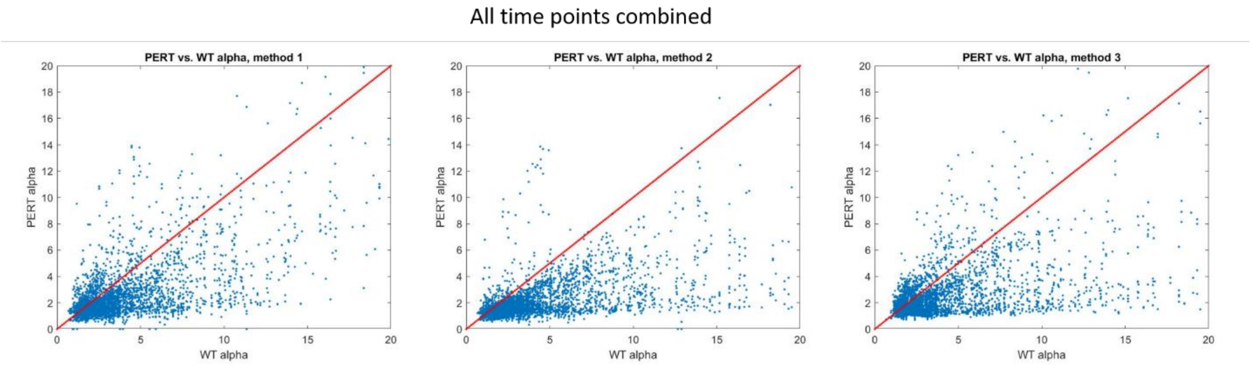

Supplementary Fig. 8: PERT alpha vs. WT alpha for all 3 perturbation methods. Source data are provided as a Source Data file.

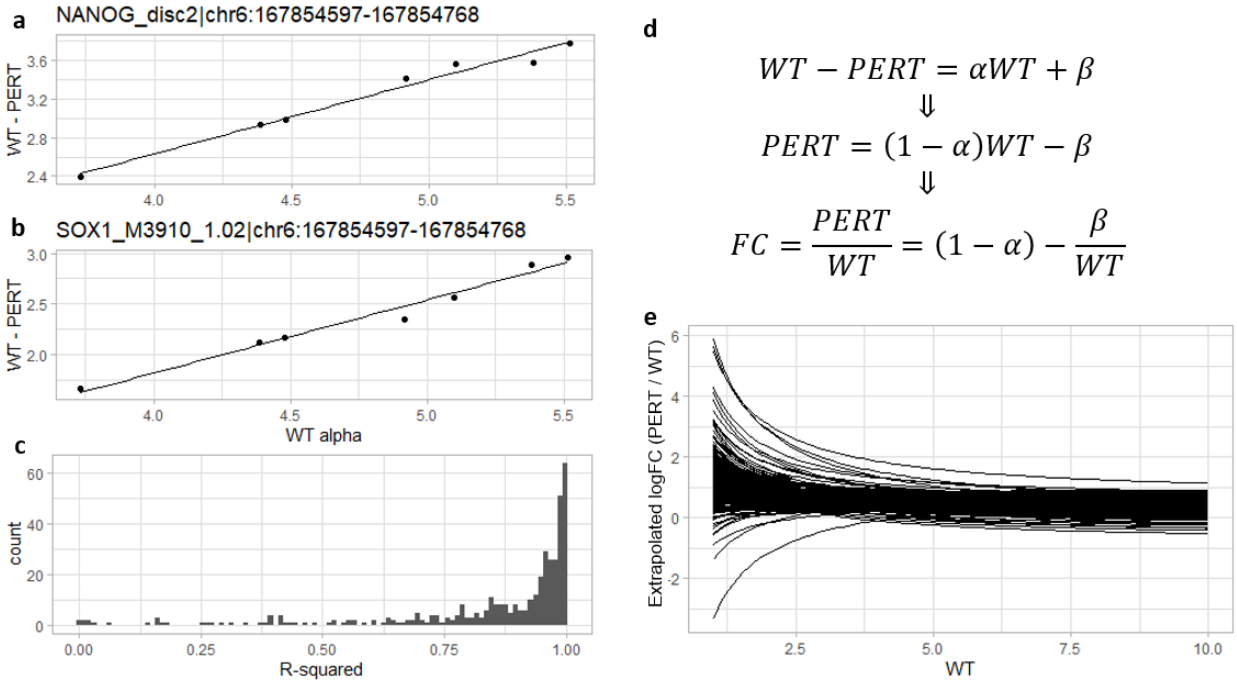

Supplementary Fig. 9: A linear relationship between the absolute perturbation effect and the WT activity levels across time. **a-b.** examples of a fitted linear regression line (WT ~ delta, where delta = WT – PERT) for two contributing FRSs in the same region. Both display a clear linear relationship with a different slope. **c.** R^2 values for all fitted models shows that the relationship is overwhelmingly linear across FRSs. **d.** transition from absolute effect as a linear function of the WT activity to the fold-change values as a function of WT activity. **e.** using the model parameters from the models fitted for each FRSs to extrapolate the log Fold-change values for that FRS as a function of WT activity. The FC decays to a constant in sufficiently high activity levels. Source data are provided as a Source Data file.

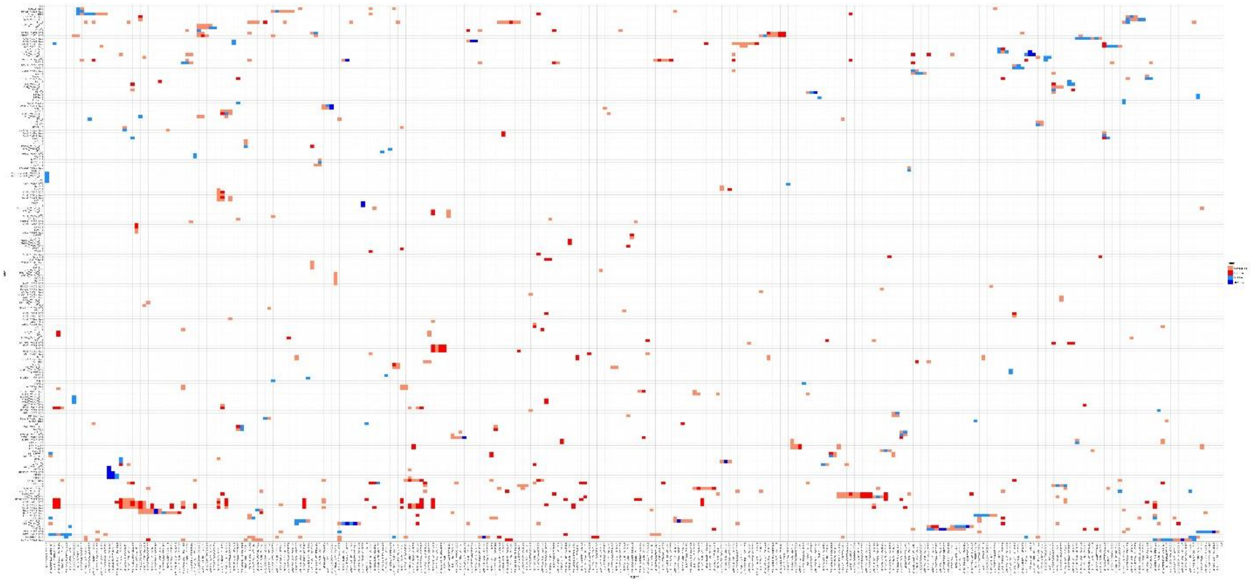

Supplementary Fig. 10: Functional FRSs heatmap, showing the perturbed motif (Y-axis) and the genomic region (X-axis). Colors correspond to the FRS category. Source data are provided as a Source Data file.

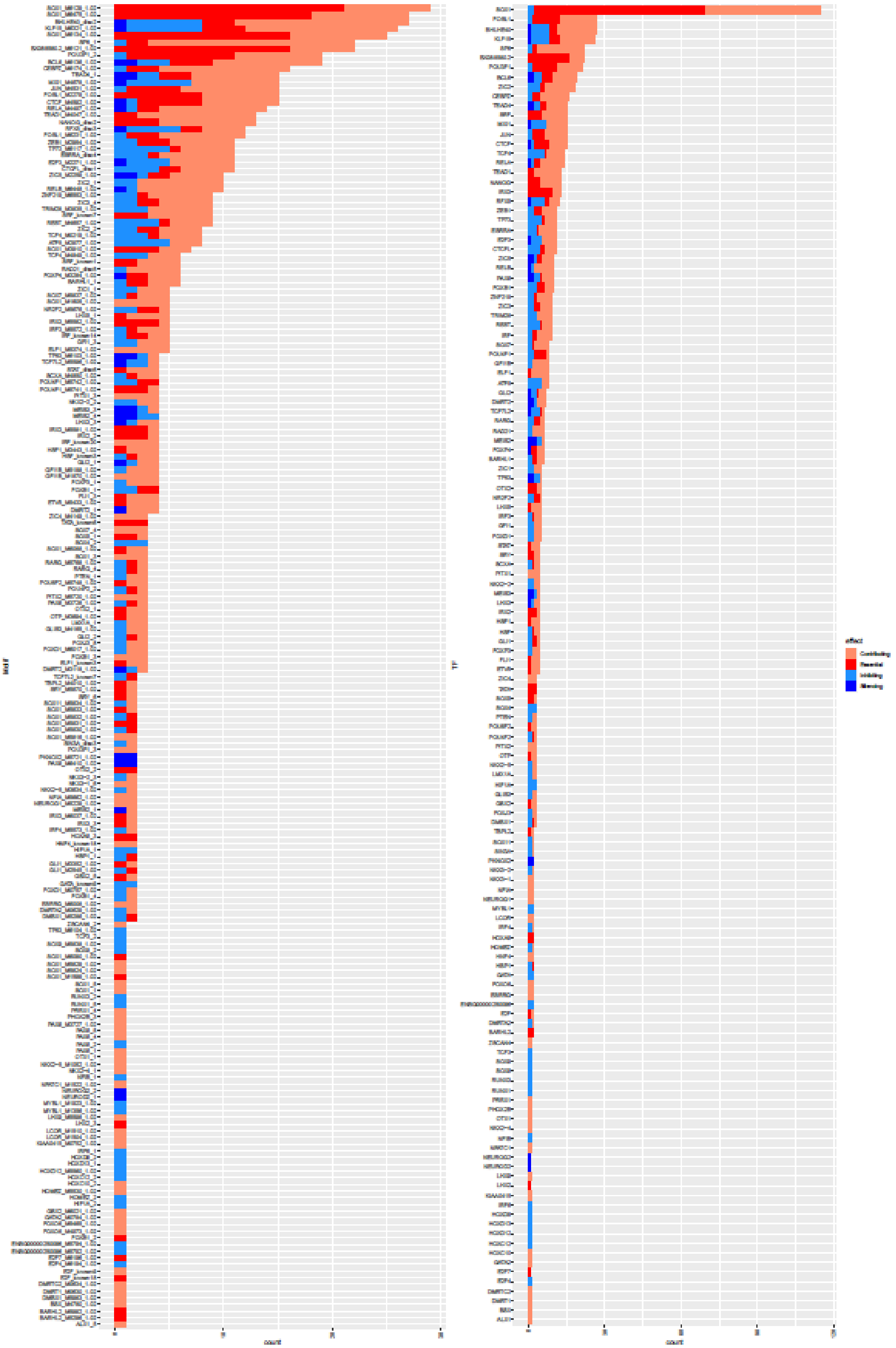

Supplementary Fig. 11: Bar plots illustrating the composition of categories for each motif (left) and aggregated across motifs for each transcription factor (right). Source data are provided as a Source Data file.

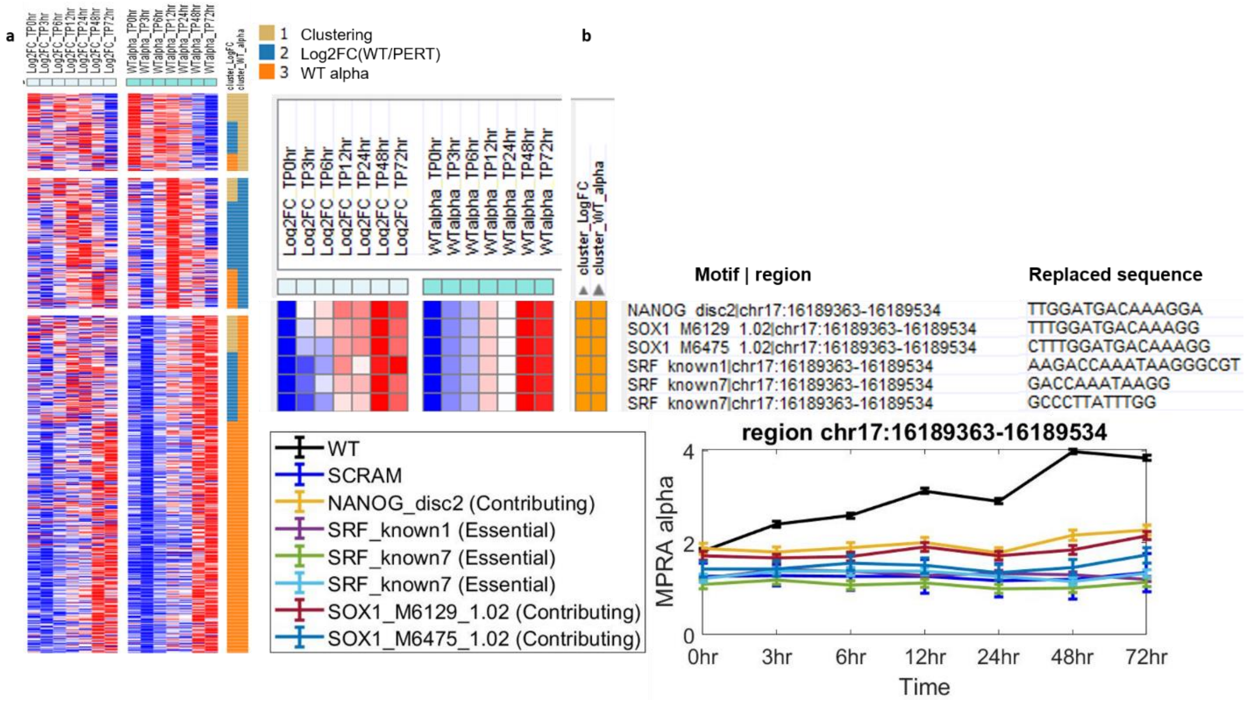

Supplementary Fig. 12: (a) Temporal clustering of FRSs signal (WT, Log2FC), clustered by the WT alpha signal and then by the Log2FC signal. Each row shows normalized values – ranged from the lowest (blue) to the highest (red). (b) Sequence specific temporal effects. The WT sequence is indicated in black, centered at the mean activity and including error bars of ±1SD across the 3 replicates, and SCRAM in blue including error bars of mean ± 1SD across all scrambled sequences. Each motif is plotted in a different color including error bars of mean ± 1SD across the 3 replicates and its perturbation effect in the regions is indicated in the text box. Source data are provided as a Source Data file. Source data are provided as a Source Data file.

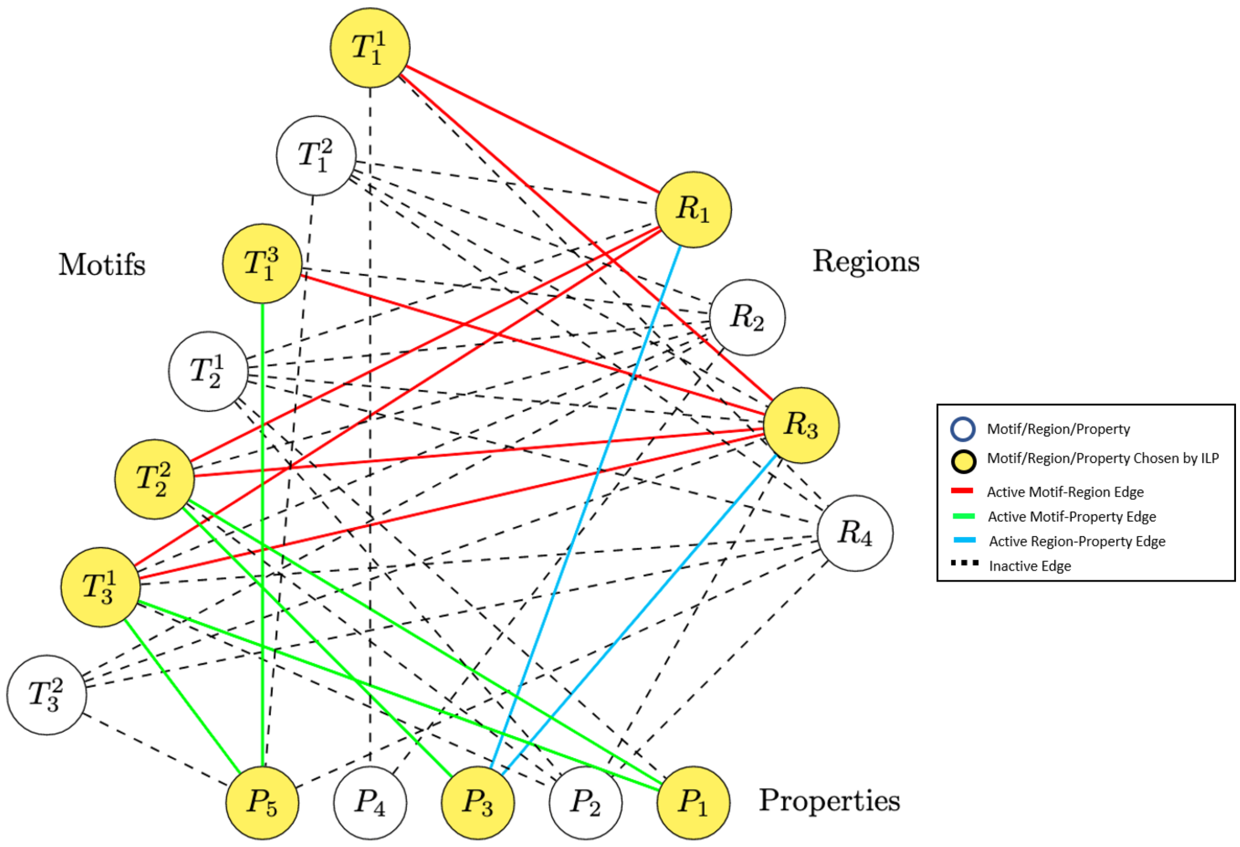

Supplementary Fig. 13: Motifs and regions selection scheme represented by a tri-partite graph.

Supplementary Tables and Legends

a.

	**Total designed**	**After preprocessing filters**	**After per time point filters**	**After temporal filter**	**After both per time point and temporal filters**	**After both per time point and temporal filters (no dup)**
**Method 1**	2144	2082	1008	1195	850	747
**Method 2**	2144	2086	1042	1227	892	775
**Method 3**	2144	2114	998	1364	915	749

b.

	**Overlap after preprocessing filters**	**Overlap after per time point filters**	**Overlap after temporal filter**	**Overlap after both per time point and temporal filters**	**Overlap after both per time point and temporal**

					**filters (no dup)**
**Methods 1-2**	2058	889	1036	703	596
**Methods 1-3**	2082	879	1049	697	573
**Methods 2-3**	2086	915	1105	746	613

Supplementary Table 1: a. The designed sequence panel filtering statistics per perturbation method. **b.** Number of the sequences that overlap between perturbation methods.

a.

	**#unique sequences**	**Only active**	**Only repressive**	**Both**
**Method 1**	747	549	190	8
**Method 2**	775	693	76	6
**Method 3**	749	582	164	3

b.

**Overlap**	**#unique sequences**	**Only active**	**Only repressive**	**Both**
**Method 1-2**	596	529	46	0
**Method 1-3**	573	469	72	0
**Method 2-3**	613	552	36	0

Supplementary Table 2: a. Sequences categories (only activators / only repressors / both) statistics. **b.** Number of the sequences that overlap between perturbation methods in each category.

a.

	**#unique motifs**	**Only active**	**Only repressive**	**Both**
**Method 1**	170	68	36	66
**Method 2**	169	108	15	46
**Method 3**	169	83	27	59

b.

**Overlap**	**#unique motifs**	**Only active**	**Only repressive**	**Both**
**Method 1-2**	148	49	5	31
**Method 1-3**	148	38	11	37
**Method 2-3**	152	59	7	25

Supplementary Table 3: a. Motif main categories (repressive/active) statistics per perturbation method. **b.** Number of the motifs that overlap between perturbation methods in each category.

a.

	**#unique regions**	**Only active**	**Only repressive**	**Both**
**Method 1**	317	166	74	77
**Method 2**	306	236	34	36
**Method 3**	302	173	53	76

b.

**Overlap**	**#unique regions**	**Only active**	**Only repressive**	**Both**
**Method 1-2**	265	146	14	16
**Method 1-3**	267	115	27	34
**Method 2-3**	263	149	10	17

Supplementary Table 4: a. Region main categories (repressive/active) statistics per perturbation method. **b.** Number of the regions that overlap between perturbation methods in each category.

	Activators		Repressors	
	**Essential**	**Contributing**	**Silencers**	**Inhibitors**
**Method 1**	181	368	34	164
**Method 2**	264	429	23	59
**Method 3**	174	408	27	140

Supplementary Table 5: Sequence sub-categories (Activators: essential, contributing. Repressors: silencers, inhibitors) statistics per perturbation method.

Supplementary References

1 Inoue, F., Kreimer, A., Ashuach, T., Ahituv, N. & Yosef, N. Identification and Massively Parallel Characterization of Regulatory Elements Driving Neural Induction. *Cell stem cell* **25**, 713-727 e710, doi:10.1016/j.stem.2019.09.010 (2019).
