# Supplementary material for: Massively parallel reporter perturbation assays uncover temporal regulatory architecture during neural differentiation
Source: Nat Commun. 2022 Mar 21;13:1504. doi: 10.1038/s41467-022-28659-0 (PMC8938438; doi:10.1038/s41467-022-28659-0)
Supplement: Supplementary file 3 — Description of Additional Supplementary Files [file 41467_2022_28659_MOESM3_ESM.docx]

**Description of Additional Supplementary Files**

**Title: Supplementary Dataset 1**

**Description:** Annotation of sequences per perturbation method (pertX_pass) that passed the 4 filters and for FRSs. Each tab contains information on sequences that passed all four filters in perturbation methods 1, 2, 3 or FRSs. The columns are as follows:

motif – motif name, region: region, motif_effect – 1:essential, 2: contributing, 3: silencing, 4: inhibiting, start – motif start position within the tested sequence, end – motif end position within the tested sequence, strand – motif strand, replaced_seq – the actual replaced motif

sequence, original_cluster_WT – temporal MPRA cluster 1-4 from Inoue et al. 2019,

cluster_LogFC - temporal Lof2FC cluster, cluster_WT_alpha - temporal alpha for WT cluster, closest_genes – closest genes within 1 MB, PCHiC_celltype – closest genes as recorded by PCHiC in different cell types (celltype = hESC, NPC, excitatory, hippocampal, motor, astrocytes) from Song *et al.* 2019^2^, Log2FC_TP – Log2FC for the seven time points, WTalpha_TP – alpha from MPRAnalyze for the WT region across the seven time points, PERTalpha_TP - alpha from MPRAnalyze for the perturbation across the seven time points, func_TP – 0/1 indicating if the sequence is functional per time point, i.e. FDR<0.05, factor_celltype – indicates an overlap of this region with ChIP-seq of different factors (CTCF, NANOG, OTX2, PAX6, SOX2, SRF, TCF4, TRIM28) in different hESCs or ectoderm from Tsankov et *al.* 2015 ^3^.

Additionally, we have 3 tabs, one per each perturbation method (pertX_all), showing the following information for each sequence that was tested:

motif – motif name, region: region, motif_effect – 1: essential, 2: contributing, 3: silencing, 4: inhibiting, Fx – binary entry if the sequence passed filter 1-4, Filt: 1: filtered in 2: filtered out (duplicate), 3: filtered out (didn’t pass one of the 4 filters), start – motif start position within the

tested sequence, end – motif end position within the tested sequence, strand – motif strand,

replaced_seq – the actual replaced motif sequence, TPhr_K27ac – signal from ChIP-seq of H3K27ac in this region in that time point, TPhr_ATACseq – signal from ATAC-seq in this region in that time point, TPhr_RNAseq – signal from RNA-seq in that time point for the closest gene to this region, TPhr_MPRA – signal from MPRA in this region in that time point, TPhr_TF_RNAseq - signal from RNA-seq in that time point for the TF associated with the perturbed motif, Log2FC_TP – Log2FC for the seven time points, WTalpha_TP – alpha from MPRAnalyze for the

WT region across the seven time points, PERTalpha_TP - alpha from MPRAnalyze for the perturbation across the seven time points

**Title: Supplementary Dataset 2**

**Description:** Average signal effect per motif (separated for active and repressive) per each one of the seven time points. Consensus sequence is showed for all the averaged instances. Each tab contains information on motifs in sequences that passed all four filters in perturbation methods 1, 2, 3 or FRSs, separated for active and repressive motifs. 2 tabs (motif_categories, TF_categories) describing the categories distribution per motif and per TF. A tab describing the region by motif category heat-map.

**Title: Supplementary Dataset 3**

**Description:** Annotation of double perturbation sequences - per perturbation method and for IFRSs. Each tab contains information on all the designed joint perturbation sequences in perturbation methods 1, 2, 3, divided by same/diff PWM perturbations. The columns are similar to **Supplementary Dataset 1**. Each three rows represent the 2 single perturbations and the corresponding joint perturbation. The model tab contains the results of the model and indication if the joint perturbation effect is significant as described in the **Methods** section.

**Title: Supplementary Dataset 4**

**Description:** Candidate sequences for driving state specific (ESC or NPC) activity (**Supplementary Note 1**). Each tab contains information on such sequences (motif, region, sequence, additional motifs in the region) in perturbation methods 1, 2, 3 or FRSs for hESCs or NPCs.

**Title: Supplementary Dataset 5**

**Description:** Primers sequences.

**Title: Supplementary Dataset 6**

**Description:** Experimental design.
